# Supplementary material for: Omeprazole Inhibits Glioblastoma Cell Invasion and Tumor Growth
Source: Cancers (Basel). 2020 Jul 28;12(8):2097. doi: 10.3390/cancers12082097 (PMC7465678; doi:10.3390/cancers12082097)
Supplement: Supplementary file 1 [file cancers-12-02097-s001.pdf]

cancers

MDPI

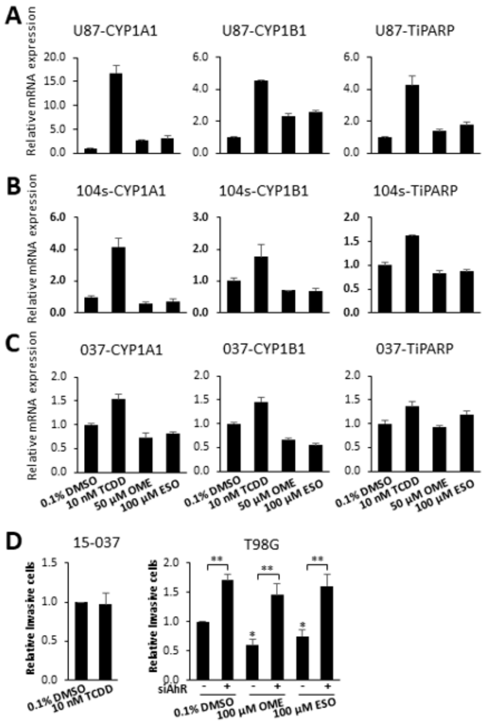

Figure S1. Effects of TCDD, OME, and ESO on induction of Ah-responsive genes. Effects of DMSO (set at 1.0), 10 nM TCDD, 50 µM OME, and 100 µM ESO on gene expression in U87-MG (**A**), 14-014s (**B**), and 15-037 (**C**) GBM cell lines were determined by real time PCR as outlined in the Methods. Effects of TCDD on 15-037 cells (**D, left**) and effects of OME and ESO on T98G cells (**D, right**) was determined in a Boyden chamber assay and results were quantitated as outlined in the Methods. Results are expressed as means ± SD for at least 3 separate determinations per treatment group and significant ($p < 0.05$) inhibition by ESO and OME (*) and reversal of these effects after AhR knockdown (**) are indicated.

Table S1. Antibody and Reagents.

Type	Name	Company	Cat #
Antibody	AhR	Enzo Life Sciences	BML-SA210
	GAPDH	Cell Signaling	5174
siRNA	Negative control	Qiagen	1027281
	siAhR	Sigma	SASI_Hs02_00332181
Chemical	4-hydroxytamoxifen	Sigma	H7904
	Flutamide	Sigma	F9397
	Leflunomide	Sigma	L5025
	Mexiletine-HCl	Sigma	M2727
	Nimodipine	Sigma	N149
	Omeprazole	Sigma	O104
	Sulindac	Sigma	S8139
	1,4-Dihydroxy-2-naphthoic acid	Sigma	281255
	Tryptamine	Sigma	193747
	Quercetin	Sigma	Q4951
	β-naphthoflavone	Sigma	N3633
	Tranilast	LKT Laboratories	T6902
	Esomeprazole potassium	LKT Laboratories	E7357

Table S2. RT-PCR Primers.

Species	Name	Forward (5′–3′)	Reverse (5′–3′)
Human	CYP1A1	GACCACAACCACCAAGAAC	AGCGAAGAATAGGGATGAAG
	CYP1B1	TATCACTGACATCTTCGGCG	ACCTGATCCAATTCTGCCTG
	CXCL12	TGGGCTCCTACTGTAAGGGTT	TTGACCCGAAGCTAAAGTGG
	CXCR4	TTTTCTTCACGGAAACAGGG	GTTACCATGGAGGGGATCAG
	MMP9	TTGGTCCACCTGGTTCAACT	ACGACGTCTTCCAGTACCGA
	TIPARP	ACACGTTCATGGCATTCAAA	TCTCAGGAGCACTTGGAAAGA
	TBP	GATCAGAACAACAGCCTGCC	TTCTGAATAGGCTGTGGGGT

Table S3. Treatment of Early Stage GBM PDX 15-037 with Omeprazole.

Treatment	Drug Route	Dose Schedule	Total Dose mg/kg	% Body Wt. Loss at Nadir	% T/C	Log Cell Kill	Tumor Free on day 35	Activity Rating
No treatment	—	—	—	0	—	—	0/5	—
Omeprazole	PO	QD3-20, 27; B.I.D: 21–26	3100	−1	41.9	0.7	0/6	Active (+)

Figure 2-B

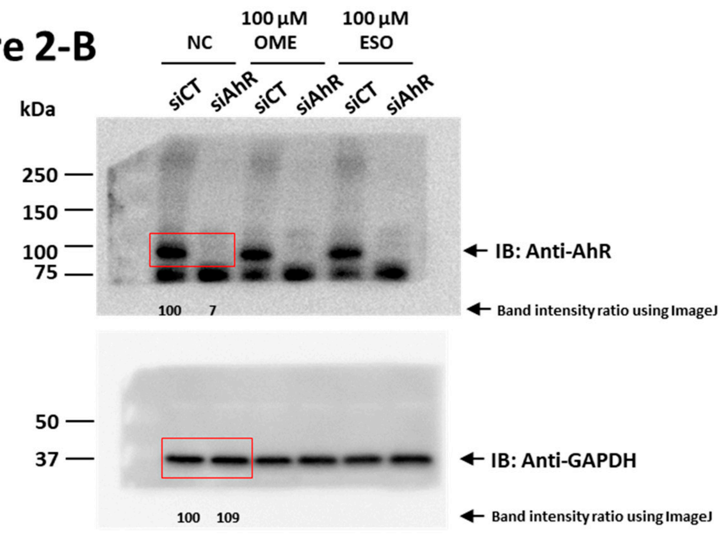

Figure 3-B

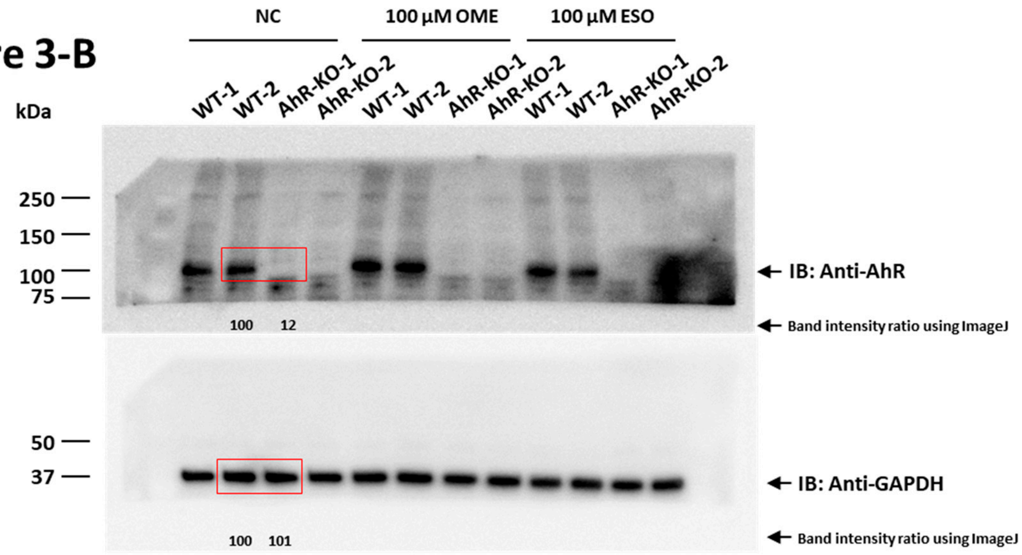

Figure 4-D

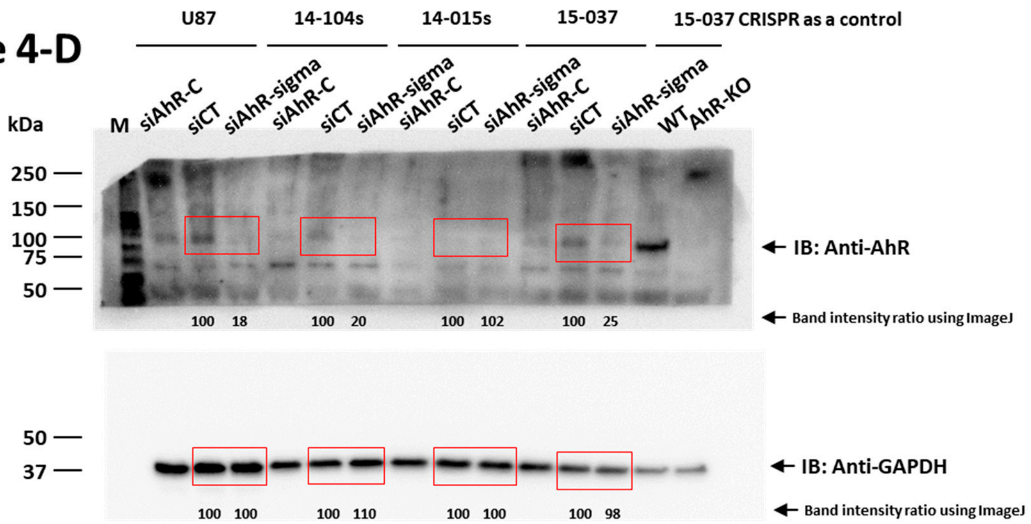

Supplemental Materials: the uncropped Western blot
